# Association of maternal constipation and risk of atopic dermatitis in offspring

**DOI:** 10.7150/ijms.96326

**Published:** 2024-07-08

**Authors:** Jyun-Yi Guo, Meng-Che Wu, Yu-Hsun Wang, James Cheng-Chung Wei

**Affiliations:** 1 Children's Medical Center, Taichung Veterans General Hospital, Taichung, Taiwan.; 2 Division of Pediatric Gastroenterology, Children's Medical Center, Taichung Veterans General Hospital, Taichung, Taiwan.; 3 Department of Post-Baccalaureate Medicine, College of Medicine, National Chung Hsing University, Taichung, Taiwan.; 4 School of Medicine, Chung Shan Medical University, Taichung, Taiwan.; 5 Department of Medical Research, Chung Shan Medical University Hospital, Taichung, Taiwan.; 6 Department of Allergy, Immunology & Rheumatology, Chung Shan Medical University Hospital, Taichung, Taiwan.; 7 Institute of Medicine, College of Medicine, Chung Shan Medical University, Taichung, Taiwan.; 8 Department of Nursing, Chung Shan Medical University, Taichung, Taiwan.; 9 Graduate Institute of Integrated Medicine, China Medical University, Taichung, Taiwan.

## Abstract

**Objectives:** Atopic dermatitis (AD) is a chronic and relapsing dermatologic disease that can affect individuals of all ages, including children and adults. The prevalence of AD has increased dramatically over the past few decades. AD may affect children's daily activities, increase their parents' stress, and increase health expenditure. Constipation is a worldwide issue and may affect the gut microbiome. Some research has indicated that constipation might be associated with risk of atopic disease. The primary objective of this retrospective cohort study was to extend and to explore the link between maternal constipation and risk of atopic dermatitis in offspring.

**Methods:** Using the Longitudinal Health Insurance Database, a subset of Taiwan's National Health Insurance Research Database, we identified 138,553 mothers with constipation and 138,553 matched controls between 2005 and 2016. Propensity score analysis was used matching birth year, child's sex, birth weight, gestational weeks, mode of delivery, maternal comorbidities, and antibiotics usage, with a ratio of 1:1. Multiple Cox regression and subgroup analyses were used to estimate the adjusted hazard ratio of child AD.

**Results:** The incidence of childhood AD was 66.17 per 1,000 person-years in constipated mothers. By adjusting child's sex, birth weight, gestational weeks, mode of delivery, maternal comorbidities, and received antibiotics, it was found that in children whose mother had constipation, there was a 1.26-fold risk of AD compared to the children of mothers without constipation (adjusted hazard ratio [aHR]: 1.26; 95% CI, 1.25-1.28). According to subgroup analyses, children in the maternal constipation group had a higher likelihood of AD irrespective of child's sex, birth weight, gestational weeks, mode of delivery, and with or without comorbidities, as well as usage of antibiotics during pregnancy. Compared to the non-constipated mothers, the aHR for the constipated mothers with laxative prescriptions <12 and ≥12 times within one year before the index date were 1.26; 95% CI, 1.24 -1.28 and 1.40; 95% CI, 1.29-1.52, respectively.

**Conclusion:** Maternal constipation was associated with an elevated risk of AD in offspring. Clinicians should be aware of the potential link to atopic dermatitis in the children of constipation in pregnant women and should treat gut patency issues during pregnancy. More study is needed to investigate the mechanisms of maternal constipation and atopic diseases in offspring.

## Introduction

Atopic dermatitis (AD) is a prevalent chronic and recurrent inflammatory skin condition that affects a substantial number of infants and children. The prevalence of AD has increased during the 21^st^ century in both developing and developed countries 1. Atopic dermatitis (AD) imposes a substantial burden on patients throughout their lifespan, affecting their social, financial, and psychological well-being, and negatively impacting their overall quality of life 2.

AD is a multifactorial disorder associated with potential genes, including genetic predisposition, environmental risk factors, dysregulated innate and acquired immune responses, as well as impaired skin barrier function 3. Clinical studies have provided evidence supporting the association between the composition of gut microbiota in early life and the development of the immune system, among other factors, in the context of AD 4. The hygiene hypothesis proposes that decreased exposure to microorganisms in early life may impact the development of allergic diseases 5. Maintaining a balanced immune response is considered a critical factor in protecting individuals who suffer from AD 6. The majority of microorganisms in the human microbiome reside in the gastrointestinal tract 7,8. The gut microbiome is believed to contribute to the development, persistence, and severity of AD through the gut-skin axis 9. The dysbiosis of the gut microbiome, along with immune system imbalance, may contribute to the development of AD 4. Microbial colonization in the body begins in earnest during the postpartum period, and this process becomes more established over time 10. Human microbial colonization begins during fetal development and continues at birth, with infancy and early childhood being recognized as crucial and sensitive periods in the establishment of the gut microbiome 9, 11. It has been shown that there are relationships among diet, nutrition, intestinal metabolism, and immune-related disease 12. The gut microbiome has been found to influence the production of short-chain fatty acids (SCFAs), which have been identified as a potential link with AD 9, 13. Interestingly, some researchers have suggested constipation might be linked to allergic disease 14, 15.

According to a systematic review, the prevalence of constipation was found to be approximately 12% in childhood and 16% in the general population worldwide, based on median estimates 16. Constipation is a common issue during pregnancy. While only a minority of individuals with constipation seek healthcare for their condition, studies have shown that people with constipation often experience impaired general health, mental health, and social functioning compared to healthy individuals 17-19. Constipation is highly associated with changes in the colonic flora, such as a decreased abundance of beneficial bacteria, such as Bifidobacteria and Lactobacilli 20. The establishment of the microbiome begins early in life and is influenced by various maternal and pregnancy-related factors, including maternal diet, genetics, mode of delivery, breastfeeding, and other environmental factors 21. Cellular leakage, including the passage of maternal IgG and microbial metabolites across the placenta, is important in the regulation of antimaternal immunity from T-cells. This mechanism is believed to be important in modulating immune responses during pregnancy 22. Mothers share their microbes and metabolites with the fetus, during delivery and lactation, through various routes 23, 24. In one study it was observed that the maternal gut microbiome serves as the primary source of transmitted strains, which have been found to persist more significantly in the infant gut 10. The relationship between maternal constipation and the risk of AD in offspring remains unclear. In this study, we hypothesized that maternal constipation may impact the risk of AD in offspring. To investigate this, we analyzed a real-world, population-based retrospective cohort from Taiwan's National Health Insurance Research Database (NHIRD).

## Methods

### Data source

The retrospective cohort study was conducted using data from Taiwan's National Health Insurance Research Database (NHIRD), a database covering more than 99% of the population of Taiwan. Claims data from 1/1/2003 to 12/31/2017 were included in the study.

The database is an invaluable resource for epidemiological research and has been used extensively 25-29. In addition, data related to basic parameters, such as patient's record, admission days, treatment, and diagnosis at discharge, can be obtained from the NHIRD. The birth certificate applications contained gestational weeks, delivery type, birth weight, single/multiple birth, stillbirth, and nationality of mother. The Maternal and Child Health Database includes parents and children. Using these databases, we were able to obtain data on the mothers' comorbidities and medications during pregnancy. The study was approved by the ethical review board of Chung Shan Medical University Hospital (approval No. CS2-21006).

### Study group and outcome measurement

This was a retrospective cohort study that enrolled 2350151 individuals from a birth certificate applications database from 2005 to 2016. After excluding children with missing data on mother's identification and nationality, as well as those with foreign nationality, there were 2082785 children in this study. The exposure group was maternal constipation. The definition of maternal constipation was a diagnosis of constipation (ICD-CM= 564.0, K59.0) with at least 3 outpatient visits or one or more admissions and use of laxatives (ATC code: A06A) one year before birth. The comparison group comprised mothers who had never been diagnosed with constipation during pregnancy. The index date was set as the birth date.

The outcome variable was defined as a diagnosis of atopic dermatitis (ICD-CM=691, L20, L22) and at least three outpatient visits times or at least one hospitalization. Both groups were followed up until the onset of atopic dermatitis, death, or 31 December 2017, whichever occurred first.

### Covariates and matching

The baseline characteristics were birth year, child's sex, birth weight (<2500; 2500-3499; ≥3500 gram), gestational weeks (<36; 36-40; ≥41 weeks), delivery (normal spontaneous delivery, NSD; cesarean section, C/S), and maternal comorbidities, including hyperlipidemia (ICD-CM= 272, E78), gestational diabetes (ICD-CM= 648.8, O99.81, O24.41, O24.42, O24.43), gestational hypertension (ICD-CM= 642.3, O13, O16.1, O16.2, O16.3), preeclampsia or eclampsia (ICD-CM= 642.4, 642.5, 642.6, 642.7, O11, O14,O15), rheumatoid arthritis (ICD-CM= 714.0, M05, M06), systemic lupus erythematosus (ICD-CM= 710.0, M32), Sjögren's syndrome (ICD-CM= 710.2, M35.0), ankylosing spondylitis (ICD-CM= 720.0, M45,M46), psoriasis (ICD- 9-CM: 6960, 6961, L40), asthma (ICD-CM= 493, J44, J45), atopic dermatitis (ICD-CM= 691, L20, L22), allergic rhinitis (ICD-CM= 477, J30), anxiety (ICD-CM= 300.0, F41), and depressive disorders (ICD-CM= 293.83, 296.2, 296.3,300.4, 311, F06.3, F32.0, F32.1, F32.2, F32.3, F32.4, F32.5, F32.9, F33.0, F33.1, F33.2, F33.3, F33.4, F33.9, F34.1). We also included pre-existing diabetes (ICD-CM= 250, E10, E11, E13, E14), hypertension (ICD-CM= 401-405, I10-I15) prior to pregnancy as covariates in the baseline characteristics. These comorbidities were defined as occurring two years before the index date and with at least three outpatient visits or one hospitalization. In addition, use of antibiotics in children during the study period was included.

Then, propensity score matching was performed by birth year, child's sex, birth weight, gestational weeks, delivery, mother's comorbidities, and antibiotic between the two groups. The propensity score was a probability that was estimated through logistic regression. The binary variable was the maternal constipation and non-maternal constipation group. By matching the propensity score, it was possible to balance the heterogeneity of the two groups.

### Statistics analysis

To compare the maternal constipation group and non-maternal constipation group, we utilized the absolute standardized differences (ASD). If the absolute standardized difference was less than 0.1, the characteristics of both groups were deemed to be similar 30. We calculated the relative risk (RR) and the 95% confidence intervals (CI) via the Poisson regression model. Kaplan-Meier analysis was conducted to determine the cumulative incidence of atopic dermatitis between the two groups. The log-rank test was used to test the significance. To assess the independent risk of the maternal constipation group, we employed the multivariate Cox proportional hazard model to estimate the hazard ratios. The statistical software was SAS version 9.4 (SAS Institute Inc., NC, USA).

## Results

**Figure 1:**
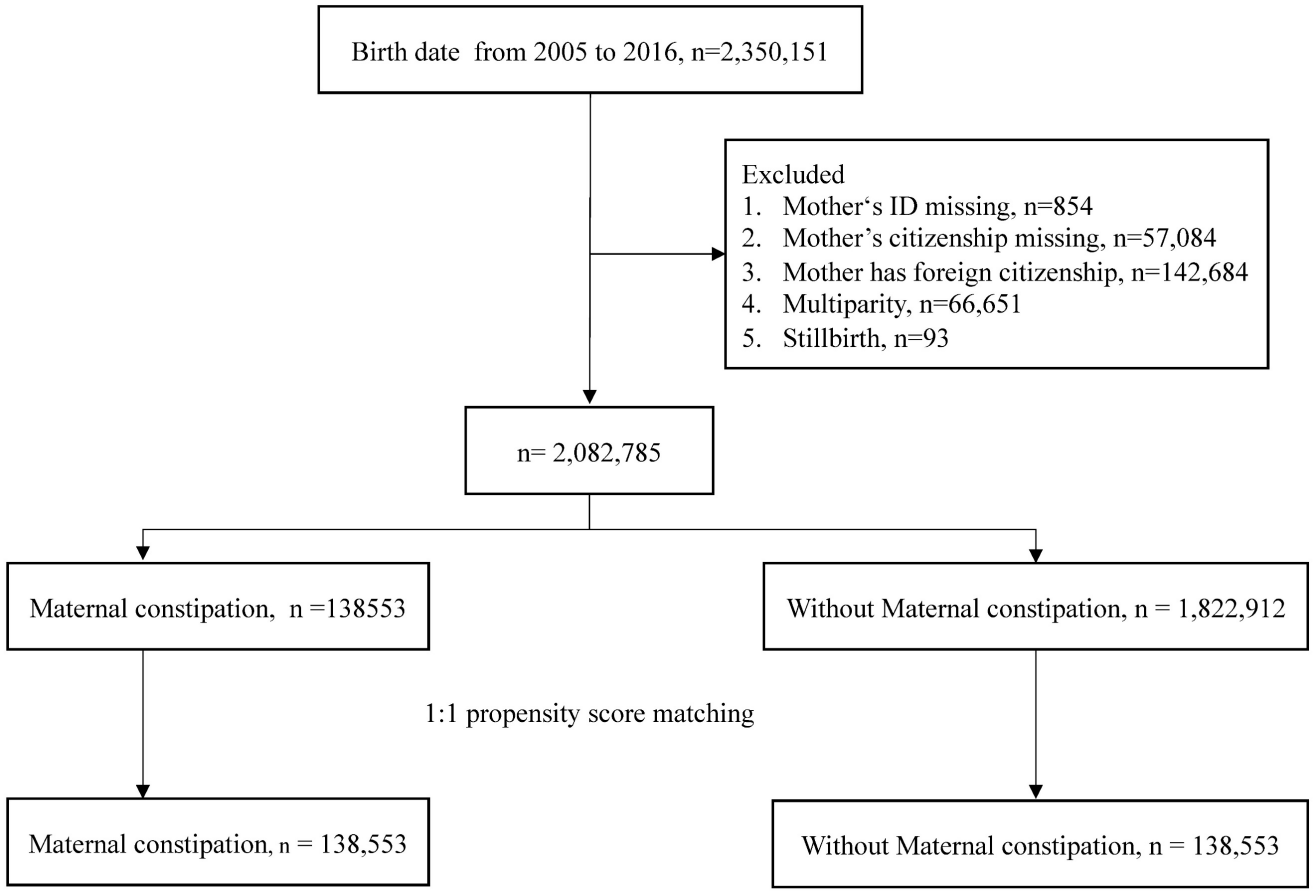
Study flow chart.

The study flow chart is shown in Fig.1. A total of 2,350,151 births were extracted from the database. The excluded data consisted of mother's ID missing, mother's citizenship missing, foreign citizenship of mother, multiparity, and stillbirth. Following exclusion of these individuals, there were 2,082,785 children left. Propensity score matching at a 1:1 ratio was used to match the maternal constipation group and the without maternal constipation group. The remaining children totaled 138,553 in each group. Table 1 shows the demographic characteristics of the constipation group and non-constipation group. There were no statistically significant differences in any of the characteristics of the children after propensity score matching. The incidence of offspring AD was 66.17 per 1,000 person-years in constipated mothers, which was higher than the rate of 53.99 per 1,000 person-years observed in the non-constipated group, as shown in Table 2. Using Poisson regression to analyze the incidence of AD, the relative risk of the maternal constipation group is 1.23-fold higher than that of the group without maternal constipation (relative risk: 1.23; 95% CI, 1.21-1.24).

Table 3 shows the Cox proportional hazard model analysis, which demonstrates that maternal constipation was 1.20-fold (aHR: 1.26; 95% CI, 1.25-1.28) higher than that of the non-constipated mothers with a p value < 0.001. In terms of other characteristics, male gender, delivery via cesarean section, mothers with Sjögren syndrome, asthma, atopic dermatitis, allergic rhinitis, anxiety, depressive disorders, and receiving antibiotics during pregnancy had greater risk of developing AD in their offspring. Subgroup analyses were performed and most of the characteristics related to maternal constipation showed a higher adjusted hazard ratio, except the rheumatoid arthritis, systemic lupus erythematosus, ankylosing spondylitis, and psoriasis subgroup (rheumatoid arthritis 1.19 95%CI 0.97-1.45, P=.0899, systemic lupus erythematosus 1.22; 95%CI 1.05-1.42, P=0.0114, ankylosing spondylitis 1.00; 95%CI 0.80-1.25, P=1, psoriasis 1.21; 95%CI 1.04-1.41, P=0.0119). In the subgroup analysis, a higher adjusted hazard ratio was noted in most of the characters along with maternal constipation (Table 4).

Comparisons with the non-constipated mothers revealed that the aHR for the constipated mothers with laxatives prescription <12 and ≥12 times within one year before the index date were 1.26; 95% CI, 1.24-1.28 and 1.40; 95% CI, 1.29-1.52, respectively (Table 5). The more severe the maternal constipation, the higher the risk of offspring AD.

**Figure 2:**
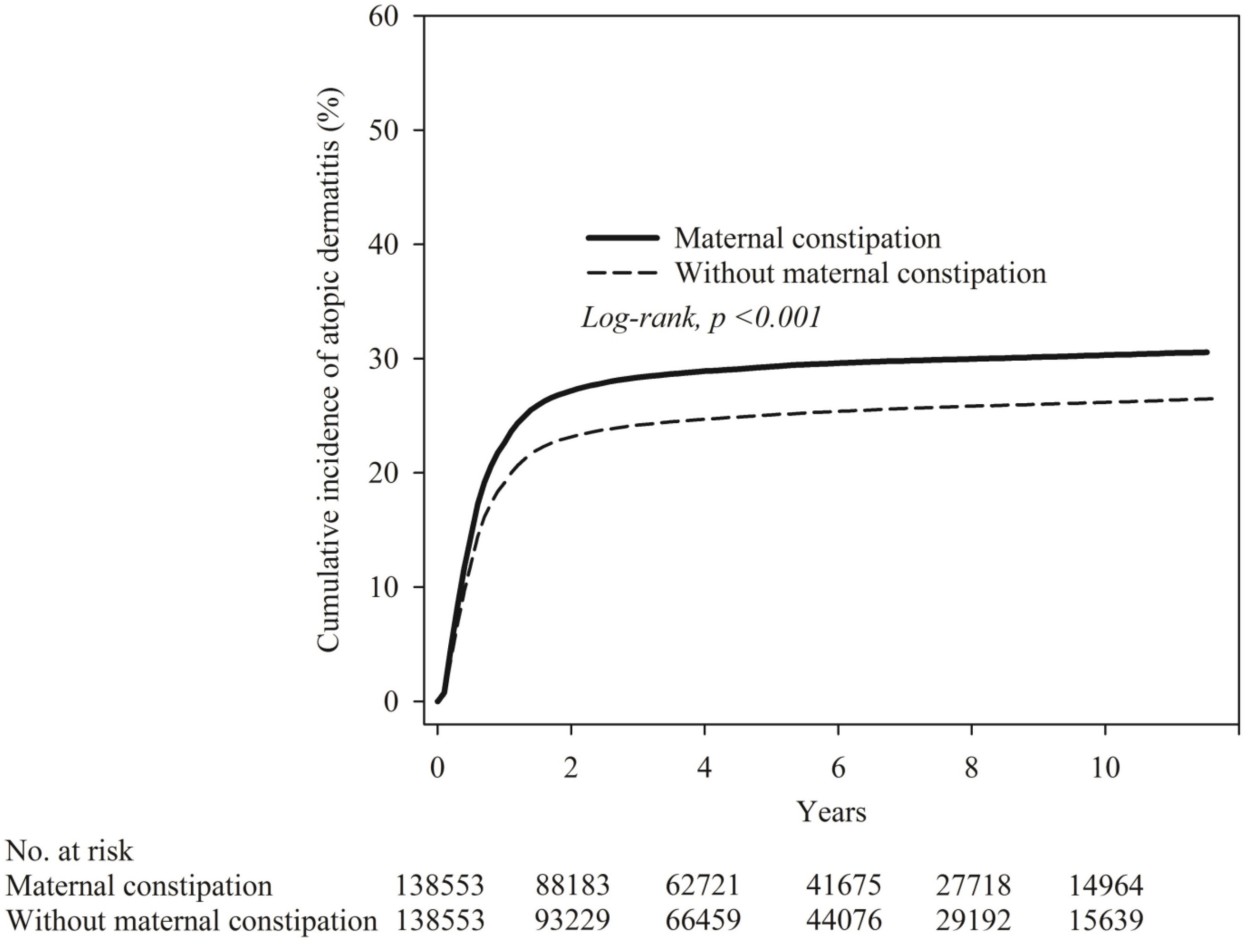
Kaplan-Meier curve of cumulative incidence proportion of atopic dermatitis in maternal constipation and maternal non-constipation groups

The Kaplan-Meier curves are shown in Figure 2. The cumulative incidence proportion of atopic dermatitis was significantly higher in the maternal constipation than in the maternal non-constipation group, and the log-rank test for the comparison of cumulative incidence curves resulted in a P value of < 0.001.

## Discussion

This longitudinal nationwide population cohort study demonstrated a significant association between maternal constipation and risk of atopic dermatitis. In this study, regardless of sex, birth weight, gestational weeks, delivery, comorbidities, and antibiotics use, maternal constipated patients had a 1.26-fold higher risk for AD than non-maternal constipated patients. This finding highlights the significance of promoting healthy bowel habits and intestinal patency to prevent constipation in pregnant mothers, which may potentially reduce the risk of AD in their children.

Our results revealed that certain characteristics of the offspring, including male gender, pre-term under 36 weeks, and birth via cesarean section, were associated with a significantly higher hazard ratio for AD. Similar findings related to sex hormones 31, delivery mode 32, 33, gestational ages have also been associated with greater risk of AD in the literature. Maternal comorbidities, including hypertension, Sjögren syndrome, anxiety, depressive disorder, asthma, allergic rhinitis, and AD have also been associated with a higher risk of AD in offspring. Several previous studies support the current findings showing that there is a correlation between maternal asthma, allergic rhinitis, pregnancy complications, and mental health conditions with their child's likelihood of developing atopic diseases 34-38. Specifically, the study showed that there is a greater risk of offspring developing atopic dermatitis when mothers use laxatives more frequently. This result implies that the greater the severity of maternal constipation, the more severe is the mother's dysbiosis, which in turn may affect the child's gut microbiome indirectly. The gut dysbiosis might not only be a causative risk factor, but also an accelerative factor in early life.

Since most studies have predominantly investigated the postpartum period in relation to the development of allergic diseases 34, 36, 39, we sought to explore whether this phenomenon could also manifest during earlier phases, such as pregnancy. The alignment between maternal and infant immunity was considered as a potential mechanism linking the maternal microbiome to the acquisition of allergic diseases in offspring 22. The composition of the perinatal gut microbiota is influenced by various factors, such as gestational age, mode of delivery, genetics, maternal microbiota, and environmental factors. During pregnancy, there is evidence of bacterial translocation, which involves the potential transfer of bacteria or bacterial products from the mother's gut to sites outside the digestive system 11, 40, 41. Additionally, studies have indicated that the maternal vaginal microbiota plays a vital role in influencing the early establishment of the infant's gut microbiota 42. Furthermore, maternal exposure to allergens can trigger an inflammatory response via cord blood IgE, which may lead to the early onset of allergic diseases in children 43-45. Signaling pathways associated with TH1 and TH17 have been demonstrated to be involved in atopic dermatitis, as well as certain autoimmune diseases, such as systemic lupus erythematosus and rheumatoid arthritis 8, 46, 47.

In atopic dermatitis, there is often an imbalance in the immune system, with an overactive TH2 response, leading to inflammation and allergic symptoms on the skin 48. SCFAs actively participate in the maintenance of the intestinal barrier and contribute to the regulation of intestinal motility 49-51. They have been shown to actively participate in promoting the expansion of regulatory T cells (Tregs) and increasing the production of IL-10. As key drivers, SCFAs contribute to the maintenance of gut homeostasis 52-55. Patients with constipation have been found to have reduced levels of some organisms (e.g., *Bifidobacterium*,* Lactobacillus*, and *Bacteroides*) compared to the healthy group. Constipation has also been linked to alterations in the structure of the intestinal flora as well as the metabolites of SCFAs 56. Gestational manipulations in the fecal microbiota are likely to promote the health of the fetus and provide the newborn with a specific microbiome 51, 57. While it is still unknown how maternal constipation changes the gut microbiome and affects the risk of developing atopic dermatitis in offspring, it might be considered a predisposing factor. Research on the microbiome of constipated mothers during pregnancy may be beneficial in preventing atopic dermatitis in children.

This study had a number of strengths. First, we utilized data from a nationwide database, which provided a larger, population-based sample than previous studies. Additionally, the longitudinal design allowed for causal inference regarding the link between maternal constipation and the development of atopic dermatitis in children. Furthermore, the study had minimal selection bias, information bias, and recall bias. Despite these advantages, there were some limitations.

Reliance on ICD-9 and ICD-10 codes may not have fully captured the complexity of the conditions being studied. Additionally, misclassification of exposure and outcome could have occurred due to the use of administrative databases. It would have been beneficial to have additional clinical data or laboratory tests to confirm the diagnosis of constipation and AD. The diagnoses of medical doctors may not have been consistent as individual expertise varies, which might have influenced the validity of the data. However, it is worth noting that any misclassifications in diagnoses were likely to be random, which could have led to an underestimation rather than an overestimation of associations. Additionally, Taiwan's NHI administration has established an ad hoc committee to monitor the accuracy of the claims data to prevent violations. Secondary, the NHIRD's lack of information on covariates such as maternal diet, personal lifestyle, family history, probiotics/prebiotics supplement or over-the-counter medication, laboratory data, or genetic survey, could have limited the ability to fully control for confounding factors in the analyses. Although we adjusted for a variety of comorbidities and medications, and used matched propensity scores to minimize potential confounders, residual unmeasured factors may have introduced bias in our results. Finally, as the majority of our patients were Taiwanese, it is unclear whether the conclusions of our study are generalizable to other ethnic groups.

## Conclusions

In conclusion, maternal constipation was associated with a 1.26-fold greater risk of AD in offspring compared with those without maternal constipation. Constipation in pregnant women or mothers should alert the physician to the increased risk of AD in their children. Gut patency issues should not be ignored in constipated mothers. Further studies are needed to determine the precise pathophysiological mechanisms linking maternal constipation and pediatric AD.

## Figures and Tables

**Table 1:** Demographic characteristics of maternal constipation group and non-constipation group

	Before PSM				After PSM			
	Without maternal constipation N = 1822912	Maternal constipationN = 138553	p value	ASD	Without maternal constipation N = 138553	Maternal constipation N = 138553	p value	ASD
**Birth year**			<0.001	0.3398			1.0000	0.0000
2005	161441 (8.86)	6197 (4.47)			6162 (4.45)	6197 (4.47)		
2006	159067 (8.73)	7574 (5.47)			7569 (5.46)	7574 (5.47)		
2007	159516 (8.75)	8388 (6.05)			8371 (6.04)	8388 (6.05)		
2008	153314 (8.41)	9337 (6.74)			9313 (6.72)	9337 (6.74)		
2009	149087 (8.18)	9986 (7.21)			9937 (7.17)	9986 (7.21)		
2010	118483 (6.50)	8989 (6.49)			8982 (6.48)	8989 (6.49)		
2011	142542 (7.82)	11811 (8.52)			11785 (8.51)	11811 (8.52)		
2012	159044 (8.72)	14857 (10.72)			14864 (10.73)	14857 (10.72)		
2013	147398 (8.09)	14391 (10.39)			14401 (10.39)	14391 (10.39)		
2014	152136 (8.35)	14691 (10.60)			14711 (10.62)	14691 (10.60)		
2015	163163 (8.95)	16752 (12.09)			16836 (12.15)	16752 (12.09)		
2016	157721 (8.65)	15580 (11.24)			15622 (11.28)	15580 (11.24)		
**Child's sex**			0.0093	0.0072			0.9879	0.0001
Female	873645 (47.93)	66904 (48.29)			66908 (48.29)	66904 (48.29)		
Male	949267 (52.07)	71649 (51.71)			71645 (51.71)	71649 (51.71)		
**Birth weight (gram)**			<0.001	0.0564			0.9902	0.0000
<2500	115992 (6.36)	8964 (6.47)			8958 (6.47)	8964 (6.47)		
2500-3499	1425360 (78.19)	110432 (79.70)			110461 (79.72)	110432 (79.70)		
≥3500	281560 (15.45)	19157 (13.83)			19134 (13.81)	19157 (13.83)		
**Gestational weeks**			<0.001	0.0000			0.5660	0.0641
<36 weeks	59530 (3.27)	4395 (3.17)			4299 (3.10)	4395 (3.17)		
36-40 weeks	1709304 (93.77)	130920 (94.49)			130997 (94.55)	130920 (94.49)		
≥41 weeks	54078 (2.97)	3238 (2.34)			3257 (2.35)	3238 (2.34)		
Delivery			<0.001	0.0644			0.8785	0.0006
NSD	1191831 (65.38)	86301 (62.29)			86340 (62.32)	86301 (62.29)		
C/S	631081 (34.62)	52252 (37.71)			52213 (37.68)	52252 (37.71)		
**Maternal comorbidity**								
Diabetes	17061 (0.94)	1590 (1.15)	<0.001	0.0208	1521 (1.10)	1590 (1.15)	0.2135	0.0047
Hypertension	16409 (0.90)	1163 (0.84)	0.0207	0.0065	1077 (0.78)	1163 (0.84)	0.0681	0.0069
Hyperlipidemia	10309 (0.57)	1237 (0.89)	<0.001	0.0385	1166 (0.84)	1237 (0.89)	0.1458	0.0055
Gestational diabetes	30617 (1.68)	2373 (1.71)	0.3551	0.0026	2305 (1.66)	2373 (1.71)	0.3160	0.0038
Gestational hypertension	6004 (0.33)	478 (0.34)	0.3284	0.0027	391 (0.28)	478 (0.34)	0.0031	0.0112
Preeclampsia or eclampsia	13301 (0.73)	943 (0.68)	0.0382	0.0059	893 (0.64)	943 (0.68)	0.2417	0.0044
Rheumatoid arthritis	1646 (0.09)	159 (0.11)	0.0038	0.0076	152 (0.11)	159 (0.11)	0.6913	0.0015
Systemic lupus erythematosus	3060 (0.17)	276 (0.20)	0.0064	0.0073	251 (0.18)	276 (0.20)	0.2757	0.0041
Sjögren syndrome	3753 (0.21)	497 (0.36)	<0.001	0.0288	463 (0.33)	497 (0.36)	0.2717	0.0042
Ankylosing spondylitis	1247 (0.07)	149 (0.11)	<0.001	0.0132	136 (0.10)	149 (0.11)	0.4410	0.0029
Psoriasis	2757 (0.15)	271 (0.20)	<0.001	0.0107	258 (0.19)	271 (0.20)	0.5716	0.0021
Asthma	21341 (1.17)	2562 (1.85)	<0.001	0.0557	2492 (1.80)	2562 (1.85)	0.3203	0.0038
Atopic dermatitis	16815 (0.92)	2095 (1.51)	<0.001	0.0538	2059 (1.49)	2095 (1.51)	0.5736	0.0021
Allergic rhinitis	126586 (6.94)	15269 (11.02)	<0.001	0.1429	15307 (11.05)	15269 (11.02)	0.8178	0.0009
Anxiety	22916 (1.26)	4889 (3.53)	<0.001	0.1490	4888 (3.53)	4889 (3.53)	0.9918	0.0000
Depressive disorders	18024 (0.99)	4142 (2.99)	<0.001	0.1437	4075 (2.94)	4142 (2.99)	0.4531	0.0029
Antibiotic	1255616 (68.880)	90055 (64.997)	<0.001	0.0826	90069 (65.01)	90055 (65.00)	0.9555	0.0002

NSD: Normal spontaneous delivery. C/S: Caesarean section.

**Table 2:** Poisson regression in incidence of atopic dermatitis

	Without maternal constipation	Maternal constipation
Follow-up duration (years)	635872	605540
Number of atopic dermatitis	34330	40071
Incidence (95% C.I.)†	53.99 (53.42-54.56)	66.17 (65.53-66.82)
Relative risk (95% C.I.)	Reference	1.23 (1.21-1.24)

† per 1000 years

**Table 3:** Cox proportional hazard model analysis for risk of atopic dermatitis in offspring

	Crude HR	p value	Adjusted HR†	p value
Maternal constipation				
No	Reference		Reference	
Yes	1.20 (1.18-1.22)	<0.001	1.26 (1.25-1.28)	<0.001
Child's sex				
Female	Reference		Reference	
Male	1.05 (1.03-1.06)	<0.001	1.12 (1.10-1.13)	<0.001
Birth weight				
2500-3499	Reference		Reference	
<2500	0.97 (0.94-1.00)	0.0352	0.94 (0.91-0.97)	0.0002
≥3500	0.99 (0.97-1.02)	0.5841	1.03 (1.01-1.05)	0.0152
Gestational weeks				
36-40 weeks	Reference		Reference	
<36 weeks	0.97 (0.93-1.02)	0.2033	1.01 (0.96-1.06)	0.8014
Post-term (≥41 weeks)	0.97 (0.92-1.01)	0.1571	1.05 (1.00-1.10)	0.0713
Delivery				
NSD	Reference		Reference	
C/S	1.07 (1.06-1.09)	<0.001	1.10 (1.08-1.11)	<0.001
**Maternal comorbidity**				
Diabetes	1.08 (1.01-1.15)	0.0249	1.05 (0.98-1.13)	0.1518
Hypertension	0.95 (0.88-1.03)	0.2388	0.90 (0.82-0.98)	0.0114
Hyperlipidemia	1.14 (1.06-1.23)	0.0004	1.05 (0.97-1.14)	0.1944
Gestational diabetes	1.03 (0.98-1.09)	0.2304	0.97 (0.92-1.02)	0.2489
Gestational hypertension	1.04 (0.91-1.18)	0.5925	1.05 (0.93-1.20)	0.4337
Preeclampsia or eclampsia	1.09 (1.00-1.19)	0.0412	1.02 (0.93-1.11)	0.7206
Rheumatoid arthritis	1.19 (0.97-1.45)	0.0899	1.17 (0.95-1.43)	0.1369
Systemic lupus erythematosus	1.22 (1.05-1.42)	0.0114	1.04 (0.89-1.22)	0.616
Sjögren syndrome	1.28 (1.15-1.43)	<0.001	1.15 (1.02-1.28)	0.0181
Ankylosing spondylitis	1.00 (0.80-1.25)	1	0.97 (0.77-1.22)	0.7878
Psoriasis	1.21 (1.04-1.41)	0.0119	1.05 (0.91-1.23)	0.4941
Asthma	1.26 (1.20-1.32)	<0.001	1.20 (1.14-1.26)	<0.001
Atopic dermatitis	1.53 (1.46-1.61)	<0.001	1.44 (1.37-1.51)	<0.001
Allergic rhinitis	1.25 (1.23-1.28)	<0.001	1.25 (1.22-1.27)	<0.001
Anxiety	1.18 (1.14-1.23)	<0.001	1.10 (1.05-1.14)	<0.001
Depressive disorders	1.14 (1.10-1.19)	<0.001	1.09 (1.05-1.14)	<0.001
Antibiotic	0.11 (0.11-0.11)	<0.001	0.11 (0.11-0.11)	<0.001

†Adjusted for sex, birth weight, gestational weeks, delivery, comorbidities, and antibiotics. NSD: Normal spontaneous delivery. C/S: Caesarean section.

**Table 4:** Subgroup analysis of the association between maternal constipation and development of atopic dermatitis in offspring

	Number of atopic dermatitis			
	Without maternal constipation	Maternal constipation	Adjusted HR	p value
**Child's sex**				
Female	16172 (24.17)	19122 (28.58)	1.28 (1.26-1.31)	<0.001
Male	18158 (25.34)	20949 (29.24)	1.25 (1.22-1.27)	<0.001
p for interaction = 0.0798				
**Birth weight (gram)**				
<2500	2118 (23.64)	2551 (28.46)	1.29 (1.22-1.37)	<0.001
2500-3499	27427 (24.83)	32004 (28.98)	1.26 (1.24-1.28)	<0.001
≥3500	4785 (25.01)	5516 (28.79)	1.25 (1.20-1.30)	<0.001
p for interaction = 0.6082				
**Gestational weeks**				
<36 weeks	1008 (23.45)	1259 (28.65)	1.31 (1.21-1.42)	<0.001
36-40 weeks	32513 (24.82)	37893 (28.94)	1.26 (1.24-1.28)	<0.001
≥41 weeks	809 (24.84)	919 (28.38)	1.26 (1.15-1.39)	<0.001
p for interaction = 0.7458				
**Delivery**				
NSD	20897 (24.2)	24448 (28.33)	1.27 (1.24-1.29)	<0.001
C/S	13433 (25.73)	15623 (29.9)	1.26 (1.23-1.29)	<0.001
p for interaction = 0.5414				
**Maternal comorbidity**				
Diabetes				
No	33931 (24.76)	39588 (28.9)	1.26 (1.25-1.28)	<0.001
Yes	399 (26.23)	483 (30.38)	1.21 (1.06-1.38)	0.0057
p for interaction = 0.4391				
Hypertension				
No	34089 (24.8)	39737 (28.92)	1.26 (1.25-1.28)	<0.001
Yes	241 (22.38)	334 (28.72)	1.35 (1.14-1.59)	0.0006
p for interaction = 0.4348				
Hyperlipidemia				
No	34018 (24.76)	39667 (28.89)	1.26 (1.25-1.28)	<0.001
Yes	312 (26.76)	404 (32.66)	1.26 (1.08-1.46)	0.0029
p for interaction = 0.8110				
Gestational diabetes				
No	33740 (24.76)	39379 (28.92)	1.26 (1.25-1.28)	<0.001
Yes	590 (25.6)	692 (29.16)	1.22 (1.09-1.36)	0.0005
p for interaction = 0.5634				
Gestational hypertension				
No	34237 (24.78)	39925 (28.92)	1.26 (1.25-1.28)	<0.001
Yes	93 (23.79)	146 (30.54)	1.39 (1.07-1.82)	0.015
p for interaction = 0.6134				
Preeclampsia or eclampsia				
No	34098 (24.77)	39780 (28.91)	1.26 (1.25-1.28)	<0.001
Yes	232 (25.98)	291 (30.86)	1.30 (1.10-1.55)	0.0029
p for interaction = 0.8520				
Rheumatoid arthritis				
No	34285 (24.77)	40020 (28.92)	1.26 (1.25-1.28)	<0.001
Yes	45 (29.61)	51 (32.08)	1.36 (0.87-2.11)	0.1738
p for interaction = 0.7114				
Systemic lupus erythematosus				
No	34252 (24.77)	39983 (28.92)	1.26 (1.25-1.28)	<0.001
Yes	78 (31.08)	88 (31.88)	0.95 (0.69-1.30)	0.7274
p for interaction = 0.0451				
Sjögren syndrome				
No	34199 (24.77)	39888 (28.89)	1.26 (1.25-1.28)	<0.001
Yes	131 (28.29)	183 (36.82)	1.40 (1.11-1.77)	0.0042
p for interaction = 0.4430				
Ankylosing spondylitis				
No	34300 (24.78)	40025 (28.92)	1.26 (1.25-1.28)	<0.001
Yes	30 (22.06)	46 (30.87)	1.55 (0.95-2.52)	0.0809
p for interaction = 0.2999				
Psoriasis				
No	34248 (24.76)	39985 (28.92)	1.26 (1.25-1.28)	<0.001
Yes	82 (31.78)	86 (31.73)	1.11 (0.81-1.52)	0.5235
p for interaction = 0.4494				
Asthma				
No	33563 (24.67)	39187 (28.82)	1.27 (1.25-1.28)	<0.001
Yes	767 (30.78)	884 (34.5)	1.20 (1.09-1.32)	0.0002
p for interaction = 0.2413				
Atopic dermatitis				
No	33585 (24.61)	39244 (28.76)	1.27 (1.25-1.28)	<0.001
Yes	745 (36.18)	827 (39.47)	1.18 (1.06-1.30)	0.0015
p for interaction = 0.1257				
Allergic rhinitis				
No	29732 (24.12)	34961 (28.36)	1.28 (1.26-1.30)	<0.001
Yes	4598 (30.04)	5110 (33.47)	1.18 (1.14-1.23)	<0.001
p for interaction = 0.0005				
Anxiety				
No	32935 (24.64)	38474 (28.78)	1.27 (1.25-1.28)	<0.001
Yes	1395 (28.54)	1597 (32.67)	1.24 (1.15-1.33)	<0.001
p for interaction = 0.5211				
Depressive disorders				
No	33212 (24.7)	38724 (28.81)	1.26 (1.25-1.28)	<0.001
Yes	1118 (27.44)	1347 (32.52)	1.26 (1.17-1.37)	<0.001
p for interaction = 0.9411				
Antibiotics				
No	25530 (52.66)	29335 (60.49)	1.25 (1.23-1.27)	<0.001
Yes	8800 (9.77)	10736 (11.92)	1.24 (1.20-1.27)	<0.001
p for interaction = 0.0405				

Adjusted for sex, birth weight, gestational weeks, delivery, comorbidities, and antibiotics NSD: Normal spontaneous delivery. C/S: Caesarean section.

**Table 5:** Cox proportional hazard model analysis for risk of atopic dermatitis in offspring

	N	No of atopic dermatitis	Crude HR	p value	Adjusted HR	p value
Laxatives frequency						
Non-constipation	138553	34330	Reference		Reference	
Maternal constipation with laxatives prescription <12 times	136967	39528	1.20 (1.18-1.22)	<0.001	1.26 (1.24-1.28)	<0.001
Maternal constipation with laxatives prescription ≥12 times	1586	543	1.51 (1.38-1.64)	<0.001	1.40 (1.29-1.52)	<0.001

Adjusted for sex, birth weight, gestational weeks, delivery, comorbidities, and antibiotics.
